# Bing-Neel syndrome successfully treated with venetoclax and zanubrutinib: A case report and review of the literature

**DOI:** 10.1097/MD.0000000000051158

**Published:** 2026-10-23

**Authors:** Shenhe Jin, Yuhui Wu, Jin Zhang

**Affiliations:** a Department of Hematology, Sir Run Run Shaw Hospital, College of Medicine, Zhejiang University, Hangzhou, Zhejiang, People’s Republic of China.

**Keywords:** Bing-Neel syndrome, venetoclax, Waldenström’s Macroglobulinemia, zanubrutinib

## Abstract

**Rationale:**

Bing-Neel syndrome (BNS) is an uncommon neurological manifestation of Waldenström’s Macroglobulinemia (WM). The diagnosis of BNS is still challenging due to its low incidence and high heterogeneity. A definite diagnosis relies on histopathology and cerebrospinal fluid (CSF) analysis to identify lymphoplasmacytic lymphocyte infiltration of the central nervous system. MYD88 L265P mutation also serves as a valuable diagnostic tool. To date, there is no consensus on the standard management of BNS, and current evidence is largely limited to reports on the use of high-dose chemotherapy and BTK inhibitors. Herein, we present a case of BNS who achieved remission following treatment with venetoclax and zanubrutinib. Furthermore, venetoclax was detected in CSF.

**Patient concern:**

A 66-year-old female with WM presenting with a headache.

**Diagnoses:**

Bing-Neel syndrome.

**Interventions:**

The patient was diagnosed with BNS through CSF examination and treated with zanubrutinib plus venetoclax.

**Outcomes:**

The patient’s symptoms resolved, and abnormal B lymphocytes were not detected in CSF.

**Lesson:**

BNS is an uncommon manifestation of WM, with significant diagnostic challenges. In the absence of standard management for BNS, high-dose chemotherapy and BTK inhibitors are widely used as front-line approaches. BCL-2 inhibitors, which have shown notable CNS-penetrating capacity, hold considerable promise for the treatment of BNS and merit further exploration.

## 1. Introduction

Waldenström’s Macroglobulinemia (WM) is a rare B-cell lymphoproliferative disorder characterized by the bone marrow infiltration of lymphoplasmacytic lymphocytes (LPLs) and a serum immunoglobulin M (IgM) paraprotein.^[1]^ Bing–Neel Syndrome (BNS) is a rare complication estimated to affect approximately 1% of all WM patients. BNS is defined as the direct infiltration of LPLs into the central nervous system (CNS), manifested by neurological symptoms such as sensorimotor deficits, visual abnormalities, and other uncommon clinical features.^[2,3]^ CNS-penetrating chemotherapy and Bruton tyrosine kinase inhibitor (BTKi) are common agents for BNS, while optimum treatment remains to be established due to limited information in the literature.^[4,5]^ Here, we present a case of BNS who achieved remission following treatment with venetoclax and zanubrutinib, and we also review the literature.

## 2. Case report

A 66-year-old female patient was diagnosed with B-cell lymphoma by lymph node biopsy at a local hospital 5 years ago, presenting with anemia, thrombocytopenia, and lymphadenopathy. Following oral administration of ibrutinib, her hemoglobin levels improved, but she was non-adherent to regular follow-up and treatment. The patient was referred to our outpatient department in December 2024 with fatigue. A blood examination showed severe anemia (hemoglobin 57g/L, normal range: 115–150g/L) and thrombocytopenia (platelet 55 × 10^9/L, normal range: 125–350 × 10^9/L). Concurrently, IgM/k-type monoclonal protein was detectable by serum immunofixation. The PET-CT scan showed high FDG metabolism in both bone marrow and multiple lymph nodes, including bilateral cervical regions, fossa axillaris, and posterior peritoneum (Fig. 1). Subsequently, bone marrow aspiration showed that lymphocytes accounted for 21.5% with morphological changes, which were recognized as abnormal B lymphocytes by flow cytometry analysis (positive for CD19, CD20, CD22, IgM, CD200, and CD79b, negative for CD5, CD10, CD38, CD138, CD23, FMC-7, CD103, and CD81, and restriction of kappa light chain). Bone marrow trephine biopsy also confirmed increased plasmacytoid lymphocytes, with immunohistochemistry results as follows: CD20 (+), CD79a (+), CD3 (−), Ki-67 (+), CD5 (−), CD43 (+), CD23 (−), BCL-2 (+), BCL-6 (−), CD10 (−), Cyclin D1 (−), MUM1 (scattered +), CD19 (+), Kappa (+), Lambda (−), CD138 (scattered +), C-Myc (10%+) (Fig. 2). MYD88 L265P mutation was also positive in bone marrow (frequency: 6.8%). In addition, axillary lymph node biopsy also indicated non-Hodgkin B-cell lymphoma with plasmacytoid differentiation, with immunohistochemistry results as follows: CD20 (+), CD79a (+), CD3 (−), Ki-67 (30% +), CD5 (−), CD43 (−), CD23 (−), BCL-2 (+), BCL-6 (−), CD10 (−), Cyclin D1 (−), SOX11 (−), MUM1 (−), CD19 (+), Kappa (++), Lambda (−), CD38 (−), and CD138 (−) (Fig. 3).

**Figure 1.**
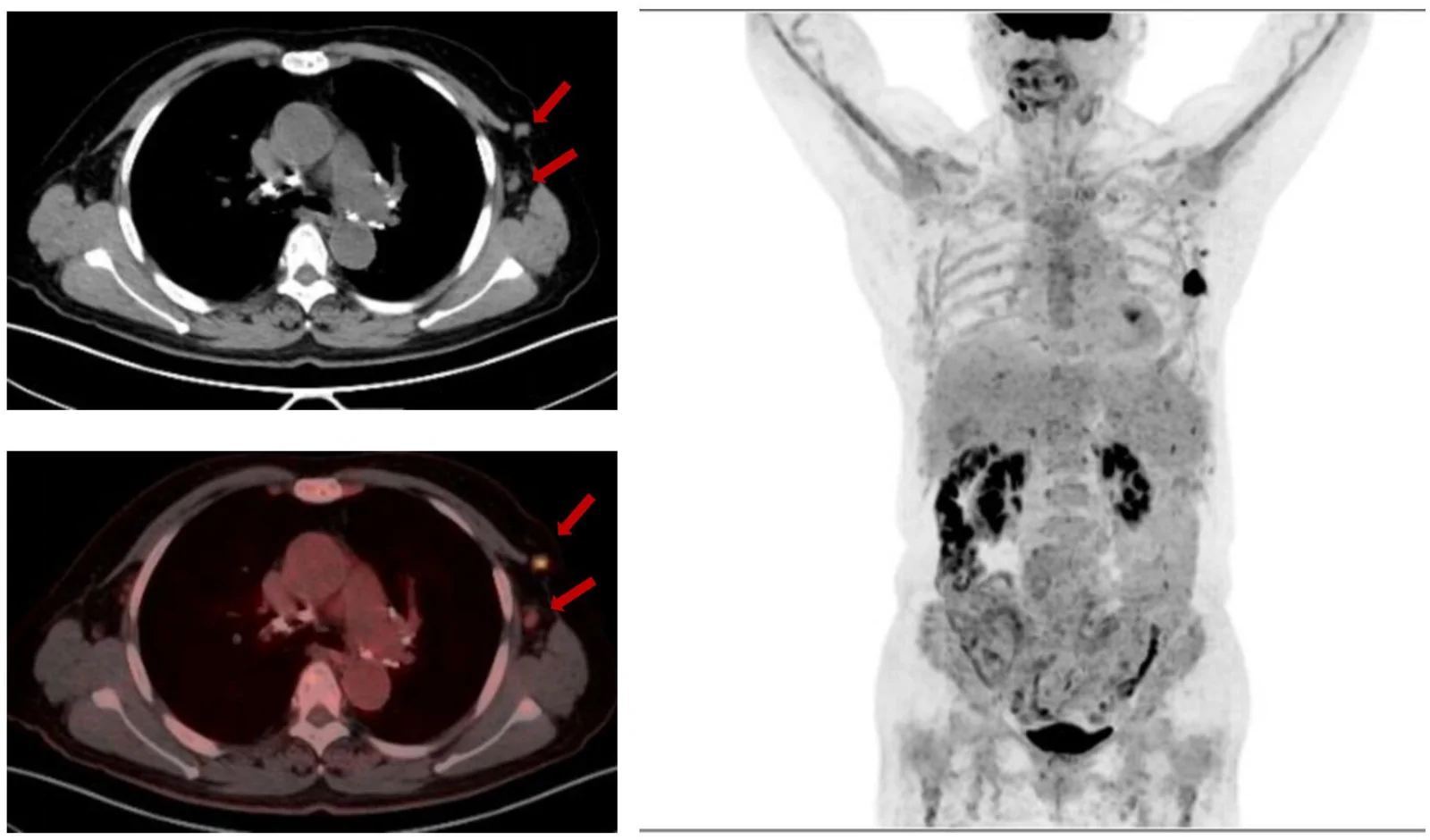
PET-CT images of patient. The FDG metabolism was elevated in bone marrow and lymph nodes of bilateral cervical regions, fossa axillaris (red arrow), and posterior peritoneum.

**Figure 2.**
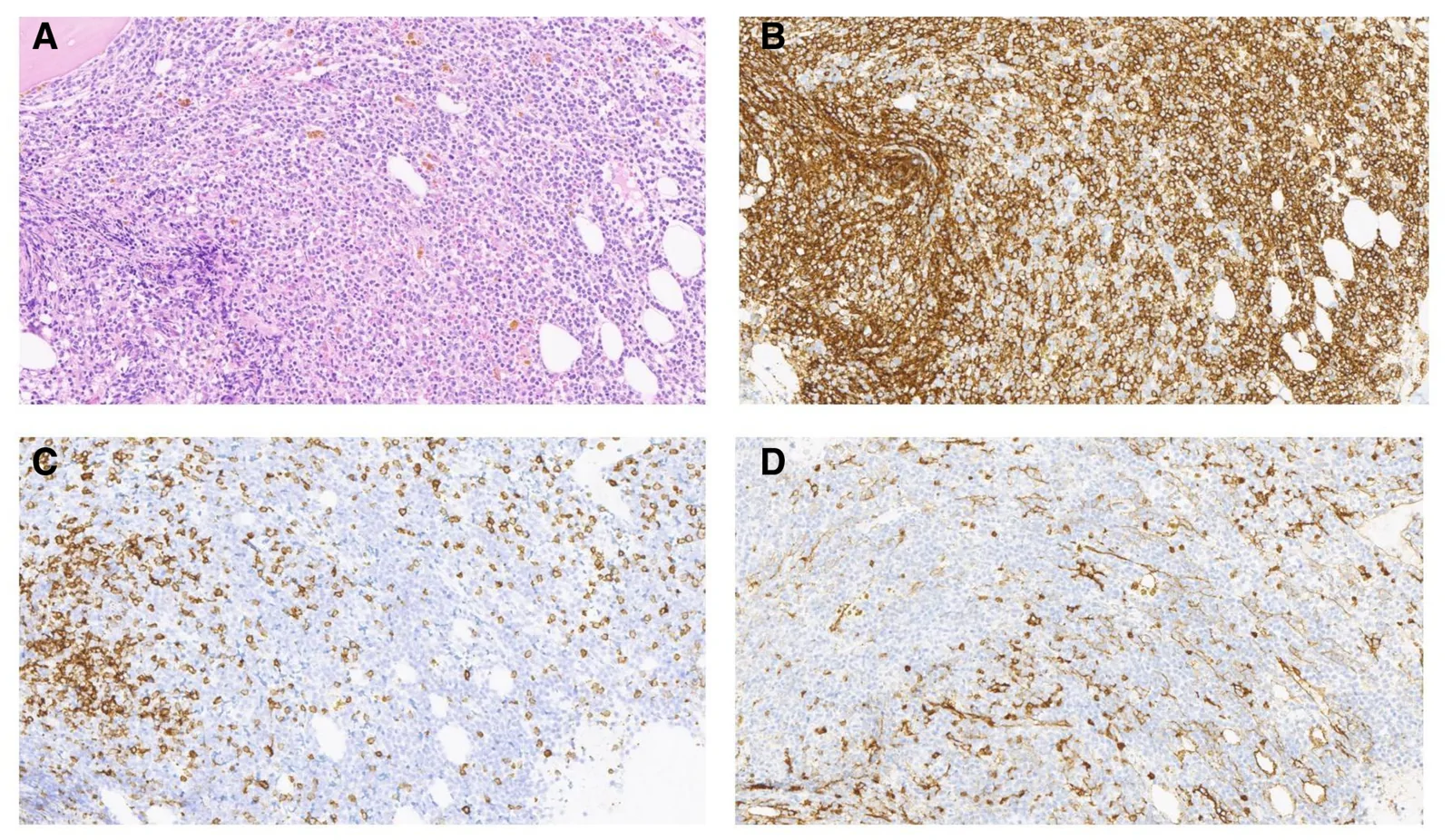
Pathology and immunohistochemistry results of bone marrow (x200) (A) H&E stain, (B) immunohistochemistry of CD20 expression, (C) immunohistochemistry of CD5 expression, (D) immunohistochemistry of CD10 expression.

**Figure 3.**
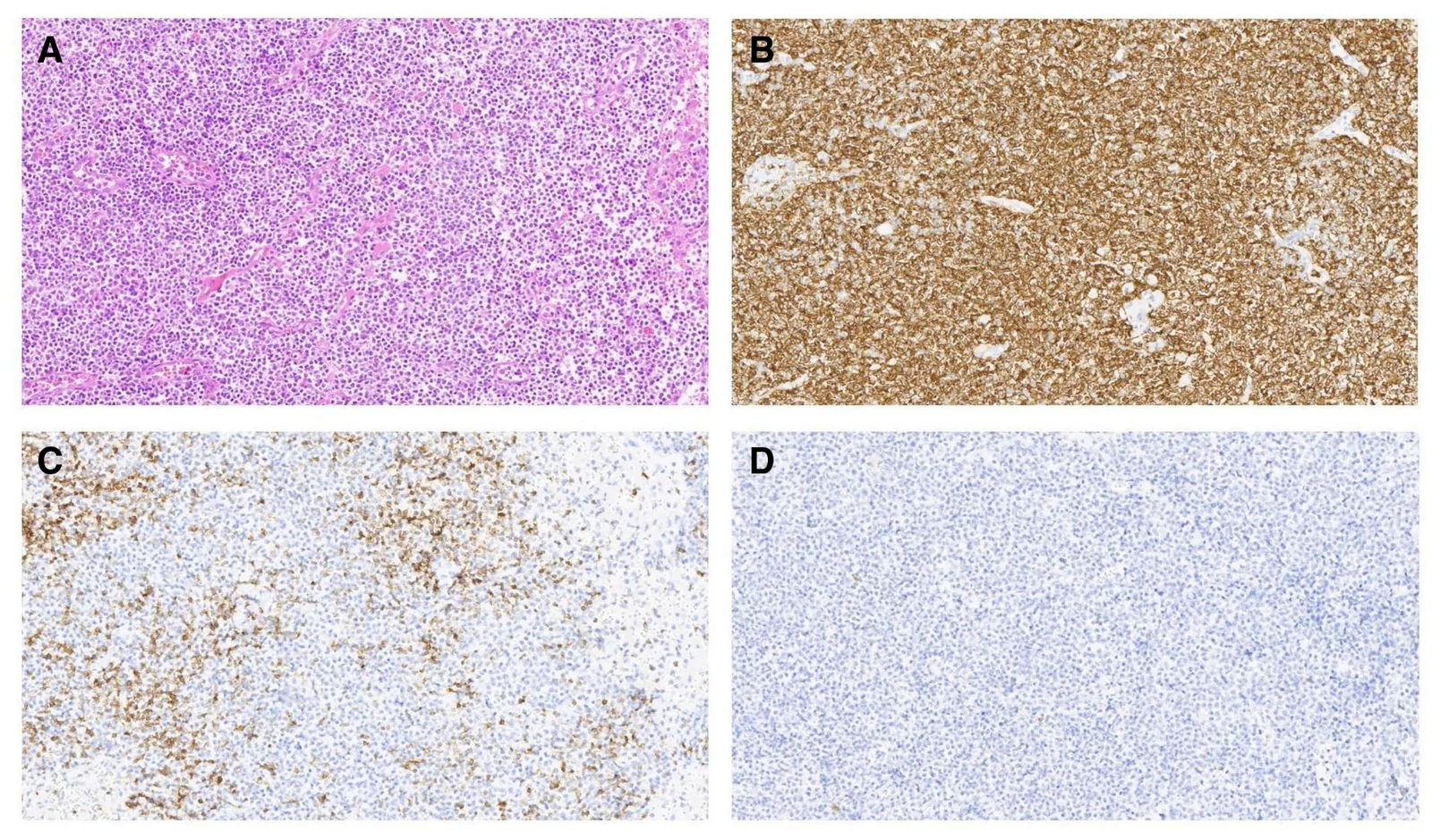
Pathology and immunohistochemistry results of axillary lymph node (x200) (A) H&E stain, (B) immunohistochemistry of CD20 expression, (C) immunohistochemistry of CD5 expression, (D) immunohistochemistry of CD10 expression.

In summary of the aforementioned examinations, the patient was diagnosed with Waldenström macroglobulinemia and received oral zanubrutinib treatment (160 mg twice daily) in February 2025. Two months after treatment, she achieved hematological improvement: hemoglobin increased from 30 to 90 g/L, platelet count from 20 × 10^9^/L to 70 × 10^9^/L, and serum monoclonal protein was undetected. However, she developed a headache. Enhanced cranial MRI was performed but only identified multiple ischemic foci. A lumbar puncture was conducted, with routine cerebrospinal fluid (CSF) examination showing a white blood cell count of 4/μl (normal range: 0–5/μl) and a moderate protein elevation (557.0 mg/L, normal range: 150–450mg/L). Additionally, flow cytometry analysis found a total of 620 cells in the CSF sample, with 38.9% of abnormal mature B lymphocytes, of small size and expressing an immunophenotype of CD19+, CD20+, CD45+, KAPPA+, CD22−, CD10−, CD38−, CD5−, CD23−, CD138−. Based on the aforementioned CSF findings, the diagnosis was considered to be Bing-Neel syndrome. In April 2025, the patient was switched to combined treatment with zanubrutinib (80 mg twice daily, dose reduced due to liver insufficiency) plus venetoclax (100 mg once daily). After 2 weeks, her headache resolved. As of the last follow-up in October 2025, the patient’s clinical and laboratory parameters remained favorable: routine blood tests revealed a hemoglobin level of 113 g/L, a platelet count of 93 × 10^9^/L, and serum monoclonal protein undetected. Meanwhile, CSF examination showed a white blood cell count of 0/μl and normalized protein. No abnormal B lymphocytes were detected by flow cytometry analysis. More importantly, venetoclax was quantified in both serum and CSF, at concentrations of 0.585 ug/ml (therapeutic threshold values: 0.5–4.0 ug/ml) and 0.042 ug/ml, respectively. This study was approved by the Ethics Committee of Sir Run Run Shaw Hospital. Informed consent was obtained from this patient.

## 3. Discussion

Bing–Neel Syndrome (BNS) is an uncommon manifestation of WM. It is believed that CNS impairment is a consequence of nerve cell demyelination, reactive gliosis, and axonal degeneration, which are triggered by LPLs infiltration and macroglobulin deposition.^[6,7]^ Clinical features of BNS are widely heterogeneous, with sensorimotor deficits, cranial nerve deficits, visual abnormalities, balance, and cognitive deficits as the most common symptoms, while some patients present with atypical symptoms, which often leads to a delay in diagnosis and poor prognosis.^[8,9]^ The definitive diagnosis of BNS depends on either a brain biopsy or CSF examination to identify the infiltration of LPLs.^[10]^ MYD88 L265P mutation was reported in 100% of BNS cases, which may also facilitate the diagnosis.^[11]^ Furthermore, radiological examination is also a valuable tool. The cranial MRI findings in BNS are primarily characterized by leptomeningeal enhancement and focal or infiltrative masses in various parts of the nervous system.^[4]^ However, due to its low incidence and high heterogeneity, the diagnosis of BNS is challenging in some cases and relies on a combination of clinical features, radiological evidence, CSF or biopsy results, and molecular alterations.

Up to now, BTK inhibitors are the preferred first-line treatment for BNS, followed by CNS-penetrating chemotherapy.^[12]^ Castillo et al reported 32 patients with BNS received high-dose methotrexate, high-dose cytarabine, alkylating agent-based chemotherapy, and intrathecal-based therapy as well as other regimens as first-line treatment; 9 patients (28%) achieved complete response (CR), and 12 (38%) achieved partial response (PR), with an estimated 3-year overall survival (OS) rate of 59%.^[11]^ Simon et al also reported 44 patients with BNS, most of them treated with cytarabine, methotrexate, and fludarabine-based regimens, and 73% of patients also received intrathecal chemotherapy (alone or in combination), 45% of patients received rituximab (alone or in combination), while 14% received autologous stem-cell transplantation (auto-HSCT) and radiotherapy. The overall response rate (ORR) was 70%, and CR was 29%; all 6 patients who underwent auto-HSCT achieved response. With a median follow-up of 4.6 years, the 5-year and 10-year OS were 71% and 59%, respectively.^[13]^ A case series of 46 patients from the United Kingdom presented that 95.6% received frontline systemic therapy, including 80% of high-dose methotrexate (MTX)–based regimens and 11% of BTKi, while BTK inhibitors were the predominant second-line treatment, accounting for 91.3% The ORR and CR were 77.3% and 22.7%, the median progression-free survival (PFS) was 89 months, and the median OS was not reached in first-line treatment. The median PFS after second-line BTKi therapy was also not reached.^[14]^ Monotherapy with ibrutinib, the first-generation BTKi, in 28 BNS patients showed that 85% and 83% had improvement of clinical symptoms and radiological abnormalities; meanwhile, the 2-year OS reached 81%.^[15]^ Zanubrutinib, a next-generation BTKi, also demonstrated durable responses with acceptable toxicity, in both treatment-naïve and previously treated patients. Sarosiek et al reported 9 BNS patients received zanubrutinib, including 5 that had prior exposure to ibrutinib, and all patients had improvement in neurological symptoms to different extents.^[16]^ Becking et al also reported that 23 of 25 (92%) patients experienced clinical improvement after zanubrutinib for 3 months. In addition, 92% (12/13) and 40% (6/15) of patients achieved radiological improvement and CNS response. The median event-free survival (EFS) and OS were not reached.^[17]^

Bcl-2 is overexpressed and plays a pro-survival role in WM. Venetoclax, a Bcl-2 antagonist, is highly active in WM patients. Castillo et al reported that the ORR was 84%, and the median PFS was 30 months for patients with previously treated WM, including those previously exposed to BTKi.^[18]^ A multicenter retrospective study of 76 patients also showed that the ORR to venetoclax monotherapy was 70%, the 2-year PFS and OS were 57% and 82%, respectively.^[19]^ Castillo et al also presented a clinical trial of combination with ibrutinib and venetoclax in 45 treatment-naïve WM patients. 42% of the patients achieved very good partial response (VGPR); the 2-year PFS and OS were 76% and 96%, respectively.^[20]^ However, reports on the treatment of BNS with venetoclax are rare.

Here, we report a female patient with WM who was diagnosed with BNS 2 months after the initiation of zanubrutinib. This patient achieved both symptomatic and CNS response with a combination of venetoclax and zanubrutinib, with acceptable toxicity and good tolerability. Furthermore, venetoclax was detected in CSF, providing direct evidence of its central CNS penetration capability, as well as its potential for BNS treatment, especially for patients who had prior exposure to BTKi.

## 4. Conclusion

BNS is a rare manifestation of WM that poses significant diagnostic challenges. Given this lack of consensus on standard management, high-dose chemotherapy and BTK inhibitors have emerged as common interventions. Meanwhile, BCL-2 inhibitors, with their demonstrated CNS-penetrating ability, offer a new direction for BNS treatment.

## Acknowledgments

The authors appreciate the patient’s consent for publication of the case. This work was supported by Zhejiang Provincial Natural Science Foundation of China (ZCLQN25H1604) and Medical and Health Research Program of Zhejiang (2025KY901).

## Author contributions

**Writing – original draft:** Shenhe Jin.

**Writing – review & editing:** Yuhui Wu.

**Supervision:** Jin Zhang.

## References

[1] Scheithauer BW Rubinstein LJ Herman MM. Leukoencephalopathy in Waldenström’s macroglobulinemia. Immunohistochemical and electron microscopic observations. J Neuropathol Exp Neurol. 1984;43:408–25. 6429288

[2] Bing J Neel A. Two cases of hyperglobulinaemia with affection of the central nervous system on a toxi-infectious basis.1. Acta Med Scand. 1936;88:492–506.

[3] Minnema MC Kimby E D’Sa S. Guideline for the diagnosis, treatment and response criteria for Bing-Neel syndrome. Haematologica. 2017;102:43–51. 10.3324/haematol.2016.147728 PMC5210231 27758817

[4] Nanah A Al Hadidi S. Bing-neel syndrome: update on the diagnosis and treatment. Clin Lymphoma Myeloma Leuk. 2022;22:e213–9. 10.1016/j.clml.2021.09.014 34674984

[5] Schep SJ Vos JMI Minnema MC. Evaluation and management of bing–neel syndrome. Hematol Oncol Clin North Am. 2023;37:777–86. 10.1016/j.hoc.2023.04.008 37258354

[6] Shimizu K Fujisawa K Yamamoto H. Importance of central nervous system involvement by neoplastic cells in a patient with Walden- ström’s macroglobulinemia developing neurologic abnormalities. Acta Haematol. 1993;90:206–8. 10.1159/000204461 8140863

[7] Fintelmann F Forghani R Schaefer PW Hochberg EP Hochberg FH. Bing- Neel syndrome revisited. Clin Lymphoma Myeloma. 2009;9:104–6. 10.3816/CLM.2009.n.028 19362988

[8] Drozd-Sokołowska J Waszczuk-Gajda A Witkowska M. Bing-neel syndrome, a rare presentation ofwaldenström macroglobulinemia—A multicenter report by the polish lymphoma research group. J Clin Med. 2022;11:4447. 10.3390/jcm11154447 PMC9369437 35956064

[9] Varettoni M Defrancesco I Diamanti L Marchioni E Farina LM Pichiecchio A. Bing-neel syndrome: illustrative cases and comprehensive review of the literature. Mediterr J Hematol Infect Dis. 2017;9:e2017061. 10.4084/MJHID.2017.061 PMC5667529 29181138

[10] Rezvani H Salari S Borhani H Mataji M Azhdari Tehrani H. Bing–Neel syndrome, a rare manifestation of WM; a case report and review of literature. Clin Case Rep. 2024;12:e9034. 10.1002/ccr3.9034 PMC11150130 38840755

[11] Castillo JJ D’Sa S Lunn MP. Central nervous system involvement by Waldenström macroglobulinaemia (Bing-Neel syndrome): a multi-institutional retrospective study. Br J Haematol. 2016;172:709–15. 10.1111/bjh.13883 PMC5480405 26686858

[12] Sarosiek S Becking AL Branagan A. Report of Consensus Panel 2 from the 12th International Workshop on the management of Bing-Neel syndrome in patients with Waldenstrom’s Macroglobulinemia. Semin Hematol. 2025;62:85–9. 10.1053/j.seminhematol.2025.04.005 40414752

[13] Simon L Fitsiori A Lemal R. Bing-Neel syndrome, a rare complication of Waldenström macroglobulinemia: analysis of 44 cases and review of the literature. A study on behalf of the French Innovative Leukemia Organization (FILO). Haematologica. 2015;100:1587–94. 10.3324/haematol.2015.133744 PMC4666335 26385211

[14] Tomkins O Khwaja J Koay S. Bing-Neel syndrome: a case series of 46 patients from the United Kingdom. Blood Adv. 2025;9:4614–7. 10.1182/bloodadvances.2025016360 PMC12455091 40644614

[15] Castillo JJ Itchaki G Paludo J. Ibrutinib for the treatment of Bing-Neel syndrome: a multicenter study. Blood. 2019;133:299–305. 10.1182/blood-2018-10-879593 30523119

[16] Sarosiek S Ramirez-Gamero A Flynn CA Treon SP Castillo JJ. Zanubrutinib for the treatment of Bing-Neel syndrome. Br J Haematol. 2025;206:1136–40. 10.1111/bjh.20016 39967276

[17] Becking AL van de Mortel JPM Tomkins O. Zanubrutinib in Bing Neel syndrome: efficacy and tolerability. Leukemia. 2025;39:1260–4. 10.1038/s41375-025-02581-y PMC12055574 40164717

[18] Castillo JJ Allan JN Siddiqi T. Venetoclax in Previously Treated Waldenström Macroglobulinemia. J Clin Oncol. 2022;40:63–71. 10.1200/JCO.21.01194 PMC8683218 34793256

[19] Sawalha Y Sarosiek S Welkie RL. Outcomes of patients with relapsed/refractory lymphoplasmacytic lymphoma/waldenström macroglobulinemia treated with venetoclax: a multicenter retrospective analysis. Blood Cancer J. 2025;15:65. 10.1038/s41408-025-01271-3 PMC12000436 40234394

[20] Castillo JJ Branagan AR Sermer D. Ibrutinib and venetoclax as primary therapy in symptomatic, treatment-naïve Waldenström macroglobulinemia. Blood. 2024;143:582–91. 10.1182/blood.2023022420 PMC10873534 37971194

